# Adsorption isotherms, kinetic and thermodynamic studies on cadmium and lead ions from water solutions using Amberlyst 15 resin

**DOI:** 10.3906/kim-2107-28

**Published:** 2021-10-05

**Authors:** Adalet TUNÇELİ, Abdullah ULAŞ, Orhan ACAR, Ali Rehber TÜRKER

**Affiliations:** Department of Chemistry, Faculty of Science, Gazi University, Ankara Turkey

**Keywords:** Cadmium, lead, Amberlyst 15 resin, adsorption, thermodynamic parameters

## Abstract

Adsorption isotherms, kinetic and thermodynamic parameters for Cd(II) and Pb(II) ions in water solutions by using Amberlyst 15 resin were performed and evaluated by utilizing solid phase extraction method with the batch system at 298, 308, and 318 K. Flame atomic absorption spectrometry was utilized for absorbance measurements of Cd and Pb in solutions. The Langmuir, Freundlich, and Dubinin–Radushkevich isotherm models, respectively were implemented to equilibrium results obtained. Experimental and theoretical monolayer adsorption capacities of resin for adsorptions of Cd(II) and Pb(II) by the Langmuir isotherm model were approximately the same and they were 120 and 116 mg/g for Cd(II) and Pb(II) ions, respectively at 318 K. Most appropriate kinetic model for adsorption of Cd(II) and Pb(II) on the resin was found as pseudo-second-order. Contact time and temperature for adsorption of analytes on the resin were optimized at 45 min and 298 K. Activation energies (*E*_a_) and thermodynamic values (ΔG°, ΔH° and ΔS°) were determined and assessed. Results showed that adsorptions of Cd(II) and Pb(II) on Amberlyst 15 were spontaneous, exothermic, and chemical ion-exchange processes.

## 1. Introduction

The development of industry and increase in population have created a rapid increase in environmental pollutions over the years. The presence of Cd and Pb metal ions in water samples can create serious environmental and human health problems because of their highly toxic effects even if at low concentrations. Some industrial areas of the use of cadmium and lead are electroplating, mining, painting, corrosion, nuclear reactor control systems, radiation protection from x-ray, battery manufacturing industries, etc. [1, 2]. Their sources are also agricultural and industrial wastewaters, rivers, lakes, dams, and seas [3]. Waters containing these metal ions are extremely unsuitable for human beings when taken by the human body. Some of the health problems caused by cadmium and lead are high blood pressure, lung and prostate cancer, anemia, the collapse of nervous and immune systems, kidney function disturbances, and brain damage [1–7]. Therefore, determinations of these metal ions from natural and wastewaters are important to protect the environment and human health.

Separation and pre-concentration methods such as liquid-liquid extraction [8–13], cloud point extraction [10–12], co-precipitation [13], adsorption by solid phase extraction (SPE) [14–19] and solid phase micro extraction [20], ion-exchange [21, 22], single drop micro-extraction [23], etc. were used for the determination of Cd and Pb when concentrations of them were below detection limits. After application of these methods, various analytical techniques such as graphite furnace atomic absorption spectrometry (GFAAS) [24–26], inductively coupled plasma mass spectrometry (ICP-MS) [27], and inductively coupled plasma optical emission spectrometry (ICP-OES) [28] and flame atomic absorption spectrometry (FAAS) were used [8, 10–12, 15–19, 21]. Among them, the SPE technique was mostly used for the separation or pre-concentration of trace metal ions, and FAAS was one of the most useful techniques used because of its high sensitivity and selectivity. Many studies have been carried out for the determination of Cd and Pb in several samples such as waters, foods, biological, and environmental samples by GFAAS and FAAS with various adsorbents [14, 16, 24]. Besides determination studies, many methods have been developed to preconcentrate Cd(II) and Pb(II) ions in the environments [18, 19, 21, 29–33]. In recoveries of Cd(II) and Pb(II) ions, macromolecular styrene divinylbenzene based synthetic materials [21, 29–31], bacteria [3, 32], yeast [33, 34], various plant shells [35, 36], seaweed [37] and nano-size materials have been used as adsorbents [15, 38, 39].

The aims of this work were to perform adsorption isotherms, kinetics, and thermodynamic parameters for Cd(II) and Pb(II) ions by utilizing Amberlyst 15 resin as an adsorbent [40–42]. Kinetics, reaction orders, adsorption capacities, contact times, and thermodynamic values (ΔG°, ΔH°, ΔS°) at 298, 308, and 318 K, respectively and *E*_a_ were determined for both Cd(II) and Pb(II) ions. Amberlyst 15 has a strong ion-exchange resin, including sulfonic acid, styrene divinylbenzene groups, and H^+^ form. The results of this study will help researchers to use the adsorbent for solid phase extraction of the analytes for analytical method development.

## 2. Materials and methods

### 2.1. Chemicals and reagents

All chemicals and reagents utilized were of analytical grade. Ultrapure water (resistivity was about 18.3 MΩ cm) was utilized in experiments. Solutions were obtained by dissolving metal salts including nitrates. HCl (37%, m/m), HNO_3_ (65%, m/m), NaOH, methanol and metal salts utilized in experiments were taken from Merck (Darmstadt, Germany). The laboratory glassware and polyethylene bottles were kept in nitric acid solution (10%, v/v) overnight, rinsed thoroughly with ultrapure water, and dried before using. Standard stock solutions of Cd(II) and Pb(II) (1000 mg/L for each) were obtained by dissolving 0.28 g of Cd(NO_3_)_2_.4H_2_O and 0.16 g of Pb(NO_3_)_2_ (Merck) in 0.1 mol/L HNO_3_ solution and poured in polyethylene bottles. Solutions of Cd(II) and Pb(II) (100 mg/L for each) and working calibration solutions (0.5 – 5.0 mg/L for each element) were prepared by diluting stock solutions before using. Nitric acid and hydrochloric acid solutions (0.1, 0.5, 1.0, and 2.0 mol/L) were prepared by diluting gently concentrated HNO_3_ and concentrated HCl with ultrapure water, respectively. The NaOH solution (0.1 mol/L) was prepared by dissolving 2.01 g NaOH in enough ultrapure water and diluted to 500 mL.

Amberlyst 15 resin, which has cross-linked styrene divinylbenzene copolymer, has been taken from Rohm and Haas (Philadelphia, USA). It has some features such as strongly sulfonic acid functional groups (-SO_3_H), ionic H^+^ structure, 0–14 workable pH range, 53 m^2^/g surface area, 300 nm average pore diameter, 400 – 500 μm particle size, 1 – 120 °C temperature range and beads physical structure [42]. The resin was washed by utilizing 1 mol/L HCl solution, methanol and ultrapure water, respectively for regeneration and to remove unreacted monomers and impurities in the structure. After drying for 4 h at 70 °C, it was utilized in experiments.

### 2.2. Instruments

Varian AAS240FS Model flame atomic absorption spectrometry (Palo Alto, USA) equipped with a deuterium lamp for background correction was utilized for determinations of Cd and Pb according to recommended conditions by the manufacturer. The operating conditions of Varian Cd and Pb lamps were 4 mA and 228.8 nm for Cd and 10 mA and 283.3 nm for Pb, respectively. Spectral band passes (0.7 nm) and acetylene/air flow rates (1.4/ 13.6 L/min) were utilized for both elements. WTW 720 model pH meter (glass electrode) (Weilheim, Germany) was utilized for all pH value measurements. A thermostatic water bath shaker (Nuve ST 402, Turkey) equipped with 150 rpm fixed speed and adjusted suitable temperatures as 298, 308, and 318 K was utilized for kinetic studies.

### 2.3. Batch adsorption studies

Batch equilibrium method was performed to determine adsorption isotherms, contact times, temperatures, initial concentrations, kinetics, and thermodynamic parameters of Cd(II) and Pb(II) ions on Amberlyst 15. Batch equilibrium studies were carried out utilizing 50 mL of 0.2 mg/L Cd(II) or 50 mL of 0.2 mg/L Pb(II) standard solution, separately in a 100 mL flask containing about 50 mg of resin. pH values of solutions were adjusted to 2–10 range utilizing 0.1 mol/L HCl and 0.1 mol/L NaOH solutions. Flasks were put onto a mechanical shaker and mixed for 120 min at adjusted temperature. The suspensions in elution solutions were filtered and concentrations of Cd(II) and Pb(II) in elutions were determined by FAAS after required dilutions. Maximum analyte mass adsorbed per mass of resin was obtained at pH = 4.0 for both Cd(II) and Pb(II) [43]. Then, the range of initial concentrations of Cd(II) and Pb(II) solutions varied from 25 to 1000 mg/L in 50 mL solutions was prepared. Each solution was poured into a 100 mL volumetric flask containing about 50 mg of resin, separately, and the pH value was adjusted to 4.0. The same procedure given above was used for all solutions and concentrations of analytes were determined by FAAS to investigate the mass of analyte ion adsorbed by the resin. Adsorbed analyte amount per unit mass of resin at equilibrium *q*_e_ (mg/g) was obtained by utilizing equation (1):


(1) $$q_{\text{e}}=\frac{({\text{C}}_{\text{o}}-{\text{C}}_{\text{e}})\cdot \text{V}}{\text{m}}$$

where, C_o_ and C_e_ are initial and equilibrium concentrations of Cd(II) and Pb(II) (mg/L), respectively. V is solution volume (L) and m is resin mass (g). For isotherm studies, all experiments were repeated three times at 298, 308, and 318 K temperatures with a range of initial concentrations of Cd(II) and Pb(II) ions in solutions as given above. Kinetic data were obtained from these three different temperatures for both Pb(II) and Cd(II) ions.

## 3. Results and discussion

### 3.1. Adsorption isotherms and capacities

**Figure 1:**
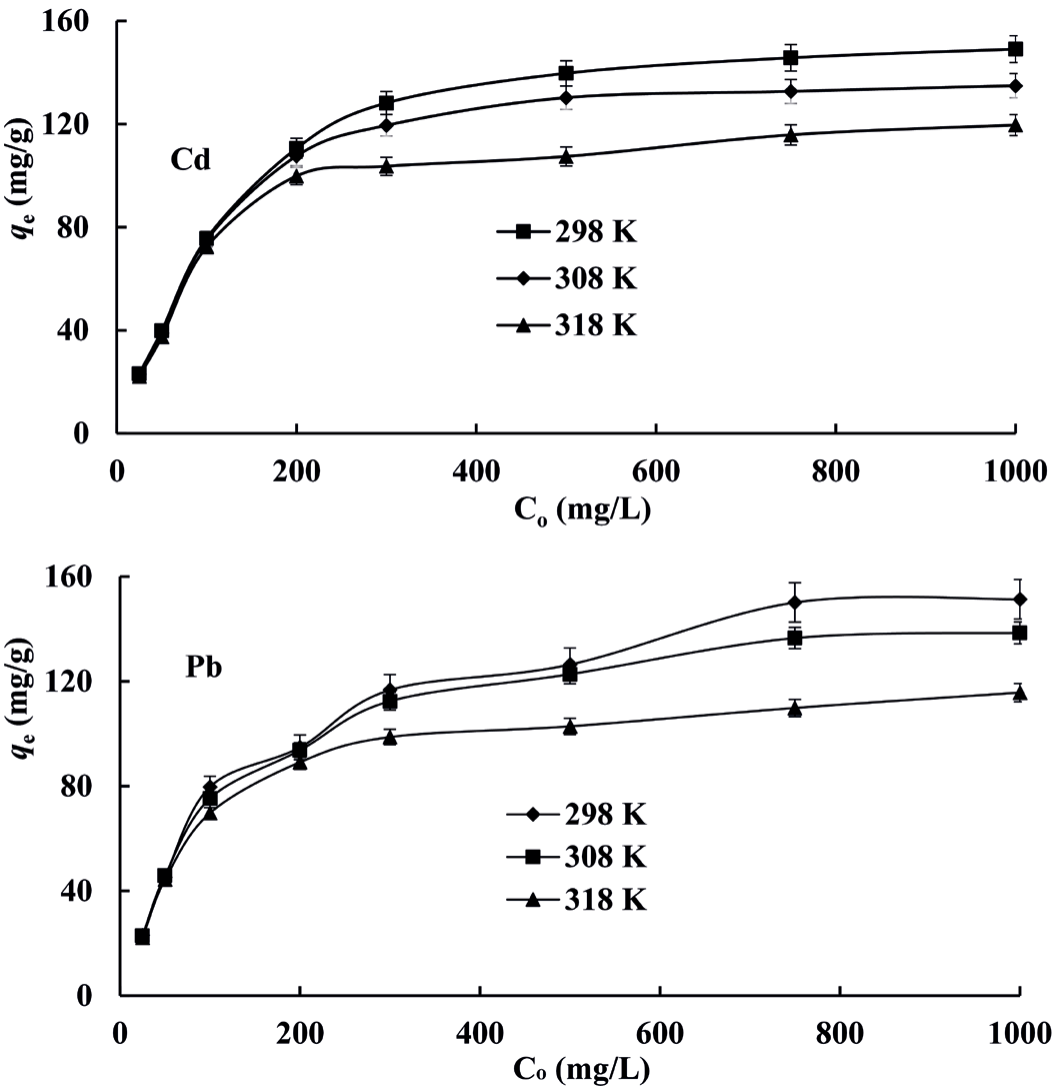
Effects of initial concentrations of Cd(II) and Pb(II) on adsorption (Sample volume: 50 mL, pH: 4).

Adsorption isotherms play important roles for adsorbents in adsorption systems, and they provide maximum adsorption capacity for metal ions from solutions. Adsorption continues until an equilibrium between concentration of analyte retained on the surface of adsorbent and concentration of analyte in solution is established [34]. A batch method was utilized to determine initial concentrations of Cd(II) and Pb(II) on the adsorption capacity of resin at pH value 4.0 and at 298, 308, and 318 K temperatures. Average values of three replicate measurements of results with standard deviation (less than 4%) were depicted in Figure 1. As seen in Figure 1, the adsorption capacity of resin is increased by increasing initial concentrations of Cd(II) or Pb(II) until the equilibrium was obtained. Initial metal concentration ensures driving force to accomplish mass transfer of ion between aqueous solution and solid phase [44]. When initial analyte concentration was increased up to approximately 400 mg/L, adsorption equilibrium capacity increased. Experimental equilibrium adsorption capacities (*q*_e_) of resin for Cd(II) and Pb(II) ions at different temperatures calculated by equation (1) were dedicated in Table 1. As seen in Table 1, they were in the range of 120–149 mg/g for Cd and 116–152 mg/g for Pb, respectively and they decreased by increasing temperature.

**Figure 2:**
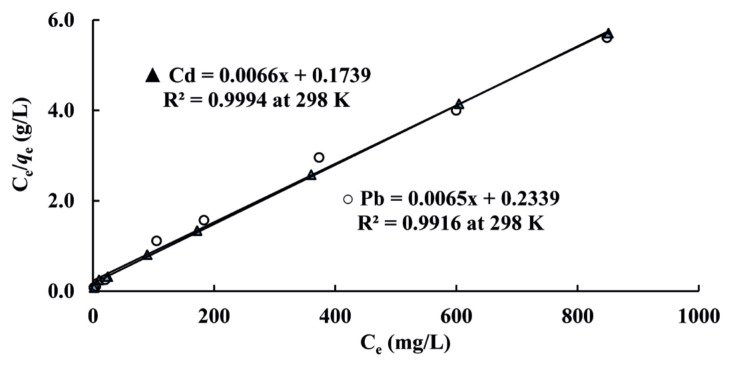
Linearized Langmuir isotherms related to adsorptions of Cd(II) and Pb(II).

The Langmuir, Freundlich, and Dubinin-Radushkevich (D-R) isotherm models were the most commonly utilized isotherms to explain adsorption equilibrium isotherms experimentally and mathematically. According to the Langmuir theory, every adsorption occurs on monolayer homogenous sites of adsorbent until saturated adsorption to be reached [1–3, 41, 44]. Linearized form of the Langmuir isotherm model was explained by utilizing equation (2):


(2) $$\frac{{\text{C}}_{\text{e}}}{q_{\text{e}}}=\frac{1}{K_{\text{L}}q_{\text{m}}}+\frac{{\text{C}}_{\text{e}}}{q_{\text{m}}}$$

where, C_e_ is the equilibrium concentration of analyte in eluent (mg/L), *q*_e_ is mass of analyte ion adsorbed per resin mass at equilibrium (mg/g), *q*_m_ is adsorption capacity of adsorbent (mg/g) and *K*_L_ (L/mg) is adsorption constant of the Langmuir isotherm model related to the free energy of adsorption. Plots of C_e_/*q*_e_ versus C_e_ values for Cd(II) and Pb(II) obtained at 298 K were depicted in Figure 2 as an example. Average values of *q*_m_ and *K*_L_ theoretically obtained from the slope (1/*q*_m_) and intercept (1/(*K*_L_*q*_m_)) by using linearized Langmuir isotherm model [44] at 298, 308 and 318 K, respectively were dedicated in Table 1. As seen in Table 1, when the temperature was increased, *q*_m_ values decreased, but, theoretically, calculated *q*_m_ values were similar with experimental *q*_e_ values for both Cd(II) and Pb(II) at 318 K. Increasing *q*_m_ value with decreasing temperature demonstrated that the adsorption process was exothermic. *K*_L_ values found for adsorptions of both Pb(II) and Cd(II) at three different temperatures were approximately the same. This means that the resin has a similar affinity towards Pb(II) and Cd(II), since *K*_L_ value is directly proportional to the binding energy [45].

In addition, dimensionless resolution factor or equilibrium parameter *(R*_L_) can be calculated to determine the suitability of the adsorption process and essential characteristics of the Langmuir isotherm model. According to the *R*_L_ value given by using equation (3), isotherm shape may be utilized to predict whether the adsorption system is preferable or nonpreferable.


(3) $$R_{\text{L}}=\frac{1}{1+K_{\text{L}}{\text{C}}_{\text{o}}}$$

where, C_o_ is initial concentrations of Cd(II) and Pb(II) (mg/L) and *K*_L_ is the Langmuir constant described above and given in Table 1. Four probabilities of the *R*_L_ values to indicate the shapes of adsorption isotherms are suitable (0 < *R*_L_ < 1), unsuitable (*R*_L_ > 1), linear (*R*_L_ = 1) or irreversible (*R*_L_ = 0) [34, 43, 45]. In this study, ranges of *R*_L_ values calculated for all initial concentrations (25–1000 mg/L) were from 0.02 to 0.51 for Cd and from 0.02 to 0.59 for Pb, respectively at 298, 308, and 318 K temperatures. These values demonstrated that adsorptions of Cd(II) and Pb(II) ions on the resin at each temperature were favorable.

The Freundlich isotherm model can be applied to heterogeneous adsorption surfaces and active sites of the resin with different energy. Using this isotherm model for the heterogeneous system is better than the Langmuir isotherm model [44, 46]. Linearized Freundlich model was explained by equation (4):


(4) $$\text{log }q_{e}=\text{log }K_{\text{F}}+(\frac{1}{\text{n}})\text{ log }{\text{C}}_{\text{e}}$$

where, *K*_F_ is adsorption capacity of resin (mg/g) and 1/n is an adsorption intensity. If 0 < 1/n < 1 - adsorption is suitable, 1/n = 0 – irreversible and 1/n = 1 – unsuitable. *K*_F_ and n values obtained from intercept and slope of the straight lines at 298, 308, and 318 K were dedicated in Table 1. As seen in Table 1, *K*_F_ values theoretically found were smaller than the values (*q*_m_) from Langmuir isotherm model and the experimental values (*q*_e_). As seen in Figure 2 and Table 1, the Langmuir model is more suitable than the Freundlich model by comparing correlation coefficients (R^2^) at 298, 308 and 318 K and monolayer adsorption of Cd(II) and Pb(II) on resin is favorable.

**Figure 3:**
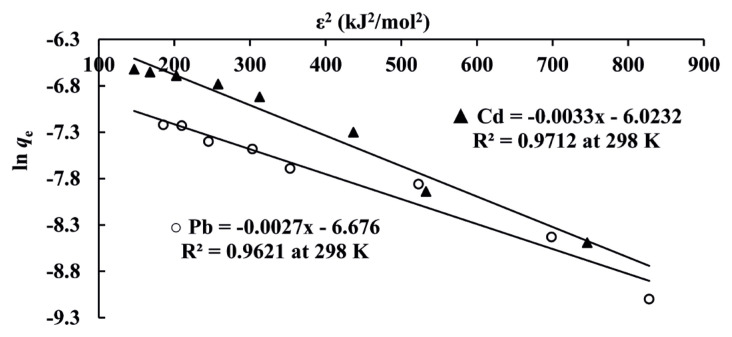
Linearized form of D-R isotherm for Cd(II) and Pb(II).

The Dubinin–Radushkevich (D - R) isotherm model was utilized to determine whether adsorption process was physical or chemical ion exchange [22, 41, 46]. Adsorption free energy (*E*) was obtained by using the D-R model. Experimental data were also implemented to the linear form of D - R isotherm model given by equation (5):


(5) $$\text{ln}q_{\text{e}}=\text{ln}q_{\text{m}}-\beta {ɛ}^{2}$$

where, *q*_e_ is mol of analyte ion adsorbed per mass of adsorbent (mol/g), *q*_m_ is adsorption capacity (mol/g), *β* is activity coefficient related to the mean free energy of adsorption (mol^2^/J^2^), and ɛ is the Polanyi potential associated with the maximum adsorption capacity at equilibrium, and it can be obtained by using equation (6):


(6) $$ɛ=RT\text{ln}(1+\frac{1}{\text{Ce}})$$

where T is temperature (K), *R* is international gas constant (8.314 J/(mol·K)) and C_e_ is metal ion concentration (mol/L) at equilibrium, respectively. Plots of ln*q*_e_ versus ɛ^2^ values for Cd(II) and Pb(II) ions obtained by equation (5) at 298 K were depicted in Figure 3 as an example. The *q*_m_ and *β* values were obtained from intercept and slope for each plot at 298, 308 and 318 K, respectively and *q*_m_ values obtained were dedicated in Table 1. As shown in Table 1, *q*_m_ values obtained from D - R isotherm model for Cd(II) and Pb(II) were reduced by increasing the temperature of the solution. This showed that the process was exothermic.

The free energy difference between the adsorbed surface of the resin and saturated liquid concentration of element ions to be adsorbed was explained as adsorption potential by the Polanyi and by Dubinin [47]. Average free energy *E* (kJ/mol) for adsorption was given by utilizing equation (7):


(7) $$E=\frac{1}{\sqrt{-2\beta }}$$

The *E* value obtained explains the information about whether adsorption mechanism is physical or chemical ion - exchange. If 8 kJ/mol ≤ *E* ≤ 16 kJ/mol, adsorption process is chemical ion – exchange. If *E* is lower than 8 kJ/mol, adsorption process is physical exchange [48]. Average adsorption free energies of Cd and Pb at 298 K, 308 K, and 318 K calculated were dedicated in Table 1. As dedicated in Table 1, *E* values found were 12.31–13.36 kJ/mol for Cd and 13.61–15.08 kJ/mol for Pb, and the mechanism of the adsorption process was chemical ion exchange.

### 3.2. Comparison of adsorption capacities of resin with literature values

Maximum adsorption capacity (*q*_m_) values of Amberlyst 15 resin for recoveries of Cd (II) and Pb (II) ions obtained from the Langmuir isotherm model (Table 1) were compared with previously reported literature values on different types of adsorbents [49–56]. They were dedicated in Table 2. As seen in Table 2, *q*_m_ values were widely different from each other because the experiments studied were carried out under different conditions such as pH value of solution, initial concentration of element, and adsorbent preparation. The *q*_m_ values of the resin for Cd (II) ion obtained at three different temperatures were within the previous studies [49, 51]. The *q*_m_ values for Pb (II) ion were similar to the result obtained from Oxidized MWCNTs adsorbent [56].

### 3.3. Chi-square (X^2^) analysis

The *X*^2^ analysis provides an important information about the suitability of the isotherm model [46, 57], and it was performed to identify a suitable isotherm model for explaining adsorptions of Cd(II) and Pb(II) ions on Amberlyst 15 resin from aqueous solutions. The mathematical explanation of the *X*^2^ analysis was given by equation (8):


(8) $$X^{2}=\Sigma \;{\left(q_{\text{e}}-q_{\text{m}}\right)}^{2}/q_{\text{m}}$$

where *q*_e_ and *q*_m_ are experimental equilibrium and theoretical equilibrium adsorption capacities calculated from the models (mg/g), respectively. If calculated data from a model are close to experimental data, *X*^2^ will be low. If they are different, *X*^2^ will be a high number. As seen in Table 1, when the temperature of the solution was increased for the Langmuir isotherm model, the difference between *q*_e_ and *q*_m_ values for both Cd(II) and Pb(II) decreased and the *X*^2^ values were near to zero. Therefore, the Langmuir isotherm model was a suitable isotherm model for the adsorption process proposed. When correlation coefficients (R^2^) of isotherm models for Cd(II) and Pb(II) ions obtained were compared, R^2^ values found from the Langmuir model were about 1.0 and higher than R^2^ values from other isotherm models.

### 3.4. Adsorption kinetics

**Figure 4:**
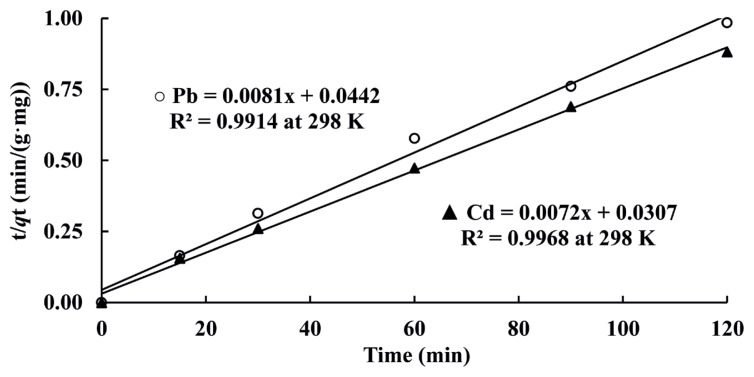
Lagergren’s pseudo-second order kinetic model for Cd(II) and Pb(II).

Orders of kinetic reactions for adsorption of Cd(II) and Pb(II) ions on Amberlyst 15 resin were investigated. For this purpose, adsorptions of initial concentrations of Cd(II) and Pb(II) on resin were studied, respectively. Nearly 400 mg/L Cd(II) and Pb(II) initial concentrations were accepted that equilibrium was reached (Figure 1). 50 mL of 400 mg/L Cd(II) or 50 mL of 400 mg/L Pb(II) solution including resin mass (about 50 mg) was mixed at time intervals (15, 30, 45, 60, 90 and 120 min) at 298 or 308 or 318 K temperatures. Concentrations of Cd(II) and Pb(II) ions in eluent solutions were determined by FAAS after filtration of the resin and the necessary dilutions. These studies were repeated three replicate measurements at 298, 308, and 318 K temperatures. Linearized forms of the Lagergren pseudo first order, pseudo-second order, and second order rate equations, respectively were implemented to results found experimentally. Rate equations can be explained by the following equations (9, 10, 11):


(9) $$\text{The pseudo first order is as follows:}\;\;\;(q_{\text{e}}-q_{\text{t}})=\text{log }q_{\text{e}}-\frac{k_{1}}{2.303}\cdot \text{t}$$

(10) $$\text{The pseudo-second order is as follows}:\;\;\;\frac{\text{t}}{q_{\text{t}}}=\frac{1}{k_{2}q_{\text{e}}^{2}}+(\frac{1}{q_{\text{e}}})\cdot \text{t}$$

(11) $$\text{The second order is as follows}:\;\;\;\frac{1}{\left(q_{\text{e}}-q_{\text{t}}\right)}=\frac{1}{q_{\text{e}}}+k\cdot \text{t}$$

where *k*_1_ is the Lagergren pseudo first-order absorption rate constant (1/min), *k*_2_ is Pseudo second order rate constant (g/(mg·min)) and *k* is second order rate constant at equilibrium (g/(mg·min)). The *q*_e_ and *q*_t_ are mass of analyte ions per mass of adsorbent (mg/g) at equilibrium and at time t (min), respectively. Plots of t/*q*_t_ values versus t were obtained and they were shown in Figure 4 as an example. Rate constants and *q*_e_ values obtained from slopes and intercepts for each plot were dedicated in Table 3. As dedicated in Table 3, most appropriate kinetic model for adsorption of Cd(II) and Pb(II) on resin was pseudo second order kinetic model [1–3] because correlation coefficients (R^2^ values: 0.9920 – 0.9971) found were highest among other models.

### 3.5. Contact time and temperature

**Figure 5:**
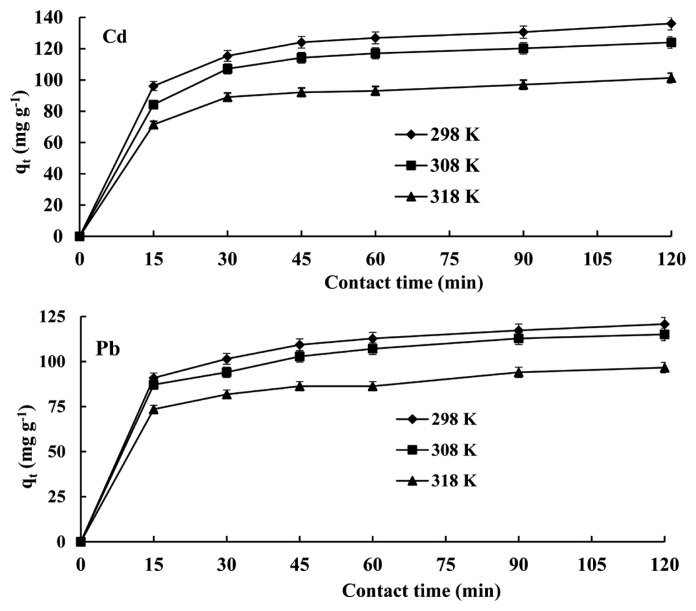
Effects of contact time and temperature for adsorption of Cd(II) and Pb(II) on Amberlyst 15 resin at pH 4.0.

Contact time and temperature for adsorption of Cd(II) and Pb(II) ions are also important for successful use of adsorbents in applications. Fast adsorption rate may be related to the form and structure of adsorbent. Thus, active adsorption sites may be located at the outer surface of the adsorbent, and mass transfer during the adsorption process may be neglected [34, 58–62]. For this purpose, effects of contact time and temperature on adsorption of Cd(II) and Pb(II) ions on resin were studied to obtain optimum contact time and temperature conditions for the adsorption process by using solutions of Cd(II) and Pb(II). Means of three replicate measurements of results with standard deviation (less than 3%) were obtained. Contact times for absorption of Cd(II) and Pb(II) ions on resin at 298, 308, and 318 K were depicted in Figure 5. As depicted in Figure 5, absorption of Cd(II) and Pb(II) ions increased by increasing contact time up to 45 min and after then it was almost constant. Therefore, the optimum contact time of analyte ions with the resin was found as 45 min. When the temperature of adsorption process by using resin was increased from 298 K to 318 K, adsorption of Cd(II) and Pb(II) decreased. These results demonstrated that the adsorption of Cd(II) and Pb(II) on resin was exothermic. By increasing the temperature of the adsorption process, the release of metal ions from the interface of adsorbent to solution might increase. The optimum temperature was observed as 298 K [34, 62].

### 3.6. Activation energies

**Figure 6:**
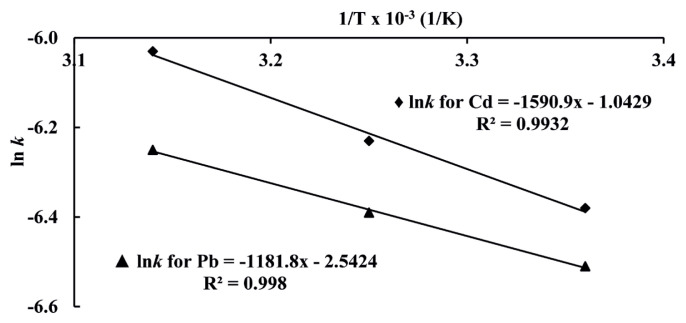
Plots of ln*k* versus 1/T for Cd(II) and Pb(II).

After determining the kinetics of ion exchange reaction related to adsorption of Cd(II) and Pb(II) ions on Amberlyst 15 resin as pseudo-second-order rate kinetics, activation energies of reactions were obtained by equation (12):


(12) $$\text{ln }k=-(\frac{E_{\text{a}}}{\text{R}})\frac{1}{\text{T}}+\text{ln A}$$

where, *E*_a_ is activation energy (J/mol), T is temperature (K) and R is gas constant. Rate constant values obtained from pseudo second order kinetic for Cd(II) and Pb(II) ions at 298, 308, and 318 K versus to 1/T were plotted and depicted in Figure 6. The activation energies of Cd(II) and Pb(II) were obtained from slops of straight lines according to equation (12) and they were given in Table 3.

### 3.7. Determination of thermodynamic parameters

Adsorption or desorption of metal ions with adsorbent may be explained by reversible reaction as metal ion in solution ⇌ metal ion on adsorbent at equilibrium.

Apparent equilibrium constant ( $K_{\text{C}}^{'}$) for adsorption based on concentration is clarified by equation (13):


(13) $$K_{\text{C}}^{\prime }=\frac{{\text{C}}_{\text{ad},\text{e}}}{{\text{C}}_{\text{e}}}$$

where, C_ad,e_ and C_e_ are equilibrium concentrations of metal ions (mg/L) on the adsorbent and in solution, respectively. Apparent equilibrium constant values ( $K_{\text{C}}^{\prime }$) were obtained by using equation (13) and C_ad,e_ and C_e_ values obtained from 50 mL of 400 mg/L Cd(II) or 50 mL of 400 mg/L Pb(II) solutions including about 50 mg Amberlyst 15 given in section 2.3. The studies were repeated three replicate measurements at 298, 308 and 318 K temperatures. Apparent equilibrium constant values ( $K_{\text{C}}^{\prime }$) versus equilibrium concentrations of Cd(II) and Pb(II) (C_e_) in solutions were plotted to obtain thermodynamic equilibrium constant ( $K_{\text{C}}^{\text{o}}$). Obtained lines were extrapolated to zero for infinite dilution and thermodynamic equilibrium constant ( $K_{\text{C}}^{\text{o}}$) values were found at 298, 308, and 318 K. The $K_{\text{C}}^{\text{o}}$ values obtained were means of three replicate measurements of Cd(II) and Pb(II) concentrations in solutions and they were given in Table 4.

**Figure 7:**
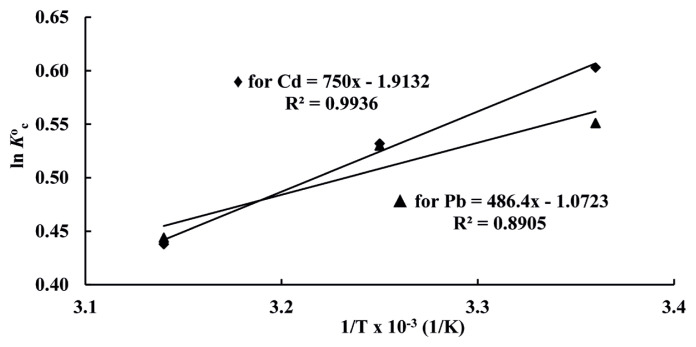
Plots of ln *K*°_C_ versus 1/T for Cd(II) and Pb(II).

Thermodynamic parameters (ΔG°, ΔH° and ΔS°) were obtained by equations (14 and 15):


(14) $$\Delta {\text{G}}^{\text{o}}=-R\text{T ln }K_{\text{C}}^{\text{o}}=\Delta {\text{H}}^{\text{o}}-\text{T}\Delta {\text{S}}^{\text{o}}$$

(15) $$\text{ln}K_{\text{C}}^{\text{o}}=\frac{\Delta {\text{S}}^{\text{o}}}{\text{R}}-\frac{\Delta {\text{H}}^{\text{o}}}{\text{RT}}$$

where, $K_{\text{C}}^{\text{o}}$ is thermodynamic equilibrium constant, *R* is gas constant, T is temperature (K), ΔG° is Gibbs standard free energy change for adsorption (kJ/mol), ΔH° is standard enthalpy change (kJ/mol) and ΔS° is standard entropy change (J/(mol·K)). ΔG° shows spontaneity of a chemical reaction. Reaction occurs spontaneously at a given temperature when ΔG° is negative. ΔS° shows disorderliness of adsorption at solid-liquid interface. Gibbs free energy changes for adsorption of Cd(II) and Pb(II) on resin were calculated by utilizing equation (14) and were dedicated in Table 4. As seen in Table 4, all ΔG° values obtained were negative. Negative ΔG° values showed that adsorptions of Cd(II) and Pb(II) ions on the resin are feasible, and adsorption is spontaneous at three different temperatures. Plots of ln $K_{\text{C}}^{\text{o}}$ versus 1/T obtained at 298 K for Cd(II) and Pb(II) ions were depicted in Figure 7. ΔH° and ΔS° changes for Cd(II) and Pb(II) were obtained from slope and intercept of each straight line according to equation (15). The ΔH° and ΔS° values found from the plots were depicted in Table 4. Negative values of enthalpy changes (ΔH°) indicated that adsorptions of Cd(II) and Pb(II) ions on resin were exothermic. Negative values of entropy changes (ΔS°) indicated that randomness between solid and liquid interfaces decreased during adsorption of Cd(II) and Pb(II) ions on Amberlyst 15.

## 4. Conclusion

Adsorption isotherms, kinetics and thermodynamic parameters for Cd(II) and Pb(II) ions from water solutions were performed by utilizing Amberlyst 15 resin in terms of its availability and adsorptive capacity. The Langmuir model was more suitable than Freundlich adsorption isotherm. Monolayer adsorption capacity of resin was found as 120 mg/g for Cd(II) and 116 mg/g for Pb(II) experimentally at 318 K. According to D-R model, Cd and Pb adsorptions on resin have been a chemical ion exchange process. The batch adsorption method depended on concentration of initial metal, pH value of solution, contact time, and temperature was utilized in studies. From kinetic data obtained, adsorptions of Cd(II) and Pb(II) ions on resin were explained by pseudo second-order kinetic. Negative values of ΔG^○^ and ΔH^○^ were confirmed that the reaction was spontaneous, exothermic, and favorable for adsorption of Cd(II) and Pb(II) ions by resin from natural and wastewaters. Negative value of ΔS^○^ indicated that the decrease occurred in randomness between solid and liquid interfaces.

## Figures and Tables

**Table 1:** The isotherm parameters of Cd(II) and Pb(II) obtained from linearized Langmuir, Freundlich, and D-R isotherms at different temperatures (T).

Element	T (K)	*q*_e_ (mg/g)	Langmuir Model			Freundlich Model			D-R Model		
			*q*_m_ (mg/g)	*K*_L_ (L/mg)	R^2^	*K*_F_ (mg/g)	n	R^2^	*q*_m_ (mg/g)	*E* (kJ/mol)	R^2^
Cd	298	149	152	0.038	0.999	22.2	3.21	0.937	273	12.31	0.971
	308	135	137	0.045	0.999	21.9	3.33	0.918	247	12.91	0.957
	318	120	121	0.039	0.999	20.3	3.67	0.892	213	13.36	0.938
Pb	298	152	154	0.028	0.992	25.9	3.60	0.934	261	13.61	0.962
	308	139	141	0.034	0.996	26.2	3.78	0.927	236	14.43	0.955
	318	116	116	0.040	0.998	24.0	3.99	0.895	197	15.08	0.935

**Table 2:** A comparison of adsorption capacities of adsorbent in this study with literature values.

Metal	Adsorbent	Adsorption capacity *q*_m_ (mg/g)	References
Cd (II)	Corn cobs supporting nano-zero valent iron	145.0	[49]
	Thiol-functionalized mesoporous silica	85.0	[50]
	Composite nano fibers membranes of poly(vinyl alcohol)/chitosan	148.8	[51]
	Surface-functionalized biochars	197.0	[52]
	Titanium-modified ultrasonic biochar	72.62	[53]
	Amberlyst 15 resin	121–152	This study
Pb (II)	oMWCNT/Ppy	26.32	[54]
	CNTs	102.04	[55]
	Oxidized MWCNTs	117.6	[56]
	Amberlyst 15 resin	116–154	This study

**Table 3:** Pseudo first order, pseudo second order and second order parameters for adsorption of Cd(II) and Pb(II) at different temperatures (T).

Element	T (K)	Pseudo first order			Pseudo second order			Second order		
		*q*_e_ (mg/g)	*k*_1_ (1/min)	R^2^	*q*_e_ (mg/g)	*k*_2_ (g/min·mg)	R^2^	*q*_e_ (mg/g)	*k* (g/min·mg)	R^2^
Cd	298	80.9	0.0324	0.8985	138.9	1.69×10^−3^	0.9968	500.0	0.0018	0.9932
	308	75.6	0.0373	0.9177	125.0	1.97×10^−3^	0.9971	95.2	0.0030	0.9839
	318	57.7	0.0318	0.8713	103.1	2.41×10^−3^	0.9967	416.7	0.0024	0.9678
Pb	298	84.0	0.033	0.8984	123.5	1.48×10^−3^	0.9914	45.1	0.0027	0.7538
	308	78.6	0.033	0.8796	120.5	1.66×10^−3^	0.9920	40.8	0.0030	0.7414
	318	63.4	0.030	0.9002	101.0	1.93×10^−3^	0.9932	147	0.0023	0.8443

**Table 4:** Thermodynamic parameters of Cd(II) and Pb(II) adsorption on Amberlyst 15 at three different temperatures.

Element	Temperature (K)		Δ*G*° (kJ/mol)	Δ*H*° (kJ/mol)	Δ*S*° (J/(mol·K))	*E*_a_ (kJ/mol)
Cd	298	1.8	−1.5			
	308	1.7	−1.4	−6.2	−16	13
	318	1.6	−1.2			
Pb	298	1.7	−1.4			
	308	1.7	−1.4	−4.1	−8.9	9.8
	318	1.6	−1.2			
